# Spatial analysis of neonatal mortality in the state of São Paulo, 2006-2010☆

**DOI:** 10.1016/j.rpped.2014.01.001

**Published:** 2014-12

**Authors:** Milena Cristina Silva Almeida, Camila Moraes Santos Gomes, Luiz Fernando Costa Nascimento

**Affiliations:** Departamento de Medicina, Universidade de Taubaté (UNITAU), Taubaté, SP, Brazil

**Keywords:** Infant mortality, Epidemiology, Geographic information systems, Spatial analysis

## Abstract

**OBJECTIVE:**

The aim of this study was to identify spatial patterns of distribution of overall, early, and late neonatal mortality rates in São Paulo state.

**METHODS:**

An ecological and exploratory study was carried in micro-regions of São Paulo sate. Mortality rates per 1,000 live births (LB) were calculated using data on overall, early, and late neonatal mortality in São Paulo between 2006 and 2010; these data were obtained from Information System and Information Technology Department of the Brazilian National Healthcare System (DATASUS). The global Moran's indices (I) were calculated for rates and thematic maps were built with these rates. Micro-regions with a high priority for intervention were identified by the box map. The software TerraView 4.2.1 was used for spatial analysis.

**RESULTS:**

The rates of early and late neonatal mortality were 6.2 per thousand LB and 2.5 per thousand LB, respectively. The global Moran's indexes (I) were I=0.13, I=0.15, and I=0.26 for overall, early, and late neonatal mortality rates, respectively; all global Moran's indices showed *p*-values <0.05. Thematic maps showed clusters of micro-regions with high rates located in the southwest and east of the state.

**CONCLUSION:**

The results presented in this study allow the implementation of policies by health managers, aiming to reduce neonatal mortality.

## Introduction

Neonatal mortality (deaths between 0 and 27 days of life) is an important health indicator of a population and accounts for approximately two-thirds of infant deaths. Is classified as early when occurring at less than 7 completed days from the time of birth, and late, when occurring after 7 completed days of age, but before 28 completed days. The neonatal mortality rate consists of early and late neonatal mortality rates, with the first representing the main component that reflects the health care provided to pregnant women in the antepartum period, at delivery, and also the care given to the newborn soon after birth and in neonatal units.^1^^,^ 2

Neonatal death is the main component of infant mortality in Brazil and was 9.7/1,000 LB in 2010, higher than in other countries such as the U.S. (4/1,000 LB), Chile (5/1,000 LB), and Canada (4/1,000 LB), among others, as reported by the World Health Organization.^3^^,^ 4 In the period between 2001 and 2010, the decrease in neonatal mortality was approximately 25%.^4^

Neonatal mortality rate can be determined by several factors, such as low and extremely low birth weight, prematurity, complex congenital malformations, and neonatal asphyxia, as well as by poor-quality prenatal care, in addition to sociodemographic factors and regional inequities.^1^^,^ 2 ^,^ 5 ^-^ 8 However, the maternal causes, the most preventable, are the most frequent underlying causes and the main triggers of neonatal mortality in developing countries.^9^^,^ 10 It is also known that most neonatal deaths occur in regions with low income, and that children born in poor regions have a higher risk of death.^10^^,^ 11

The spatial location of health events and the Geographic Information Systems (GIS) have been more frequently used in the public health area.^12^ A study on spatial analysis of neonatal death rates performed in Vale do Paraíba allowed for the identification of priority municipalities for intervention.^13^

The neonatal mortality spatial distribution analysis may provide subsidies for actions to improve health care aiming to reduce this mortality rate. Thus, the objective of the present study was to identify spatial distribution patterns of overall, early, and late neonatal mortality in the state of São Paulo during the period of 2006-2010.

## Method

This was an ecological and exploratory study with data on neonatal mortality in 63 micro-regions of the State of São Paulo, Brazil, obtained from the Department of Information and Informatics of the Unified Health System (DATASUS)^14^ in the period between 2006 and 2010. Sao Paulo is Brazil's most populous state, with approximately 41 million inhabitants. Data on live births were obtained from the Information System on Live Births (SINASC).^15^

A database was created, which included all cases of neonatal death, and the overall neonatal mortality rate was calculated per 1,000 live births, as well as the early neonatal mortality rate (that occurring at less than 7 completed days from the time of birth) and late (that occurring after 7 completed days of age but before 28 completed days), by micro-region of the state of São Paulo. The rates refer to all deaths in the neonatal period in relation to all births in the period of 2006-2010.

The public-access software program TerraView 4.2.1, developed by the National Institute of Space Research (INPE),^16^ was used for the spatial analysis of 63 micro-regions of the state of São Paulo. The spatial autocorrelation of neonatal mortality rates was estimated by global Moran's index (I).

The Moran's index is used to identify clusters of areas with similar risks for the occurrence of the outcome, and can range from -1 to +1, with values ​​close to zero indicating the absence of significant spatial autocorrelation between the values ​​of a given area and neighboring areas. Positive values indicate that the micro-regions were similar to each other, and negative values​​ indicate that the micro-regions were not similar to each other. This index is adequate for the testing of the null hypothesis, which is the spatially independent; in this case, its value would be zero. Positive values ​​(between 0 and +1) indicate direct correlation and negative values (between 0 and -1), an inverse correlation.^17^

Thematic maps of overall, early, and late mortality rates were constructed, in addition to the box map obtained through information from the Moran scatter plot,^17^ which is an additional way to visualize spatial dependence. It is divided into four quadrants: Q1 (positive values​​, positive means) and Q2 (negative values​​, negative means) indicate points of positive spatial association, in the sense that a location has neighbors with similar values​​; Q3 (positive values​​, negative means) and Q4 (negative values, positive means) indicate points of negative spatial association, in the sense that a location has neighbors with different values​​. 

In the box map, the microregions located in Q1 require special attention in order to decrease the rates of the studied outcome; in this case, neonatal mortality. The micro-regions located in Q2 have low priority of attention, as they have lower rates.^17^

The significance level (α) was set at 5%. This project was approved by the Research Ethics Committee of Universidade de Taubate, No. 045/11.

## Results

In the period from 2006 to 2010 a total of 3,000,158 live births were recorded in the state of São Paulo; there were 18,448 early neonatal deaths (6.2/1,000 LB) and 7,510 late neonatal deaths (2.5/1,000 LB). Table 1 shows the values of the overall, early, and late neonatal mortality rates in the micro-regions of São Paulo.

**Table 1:**
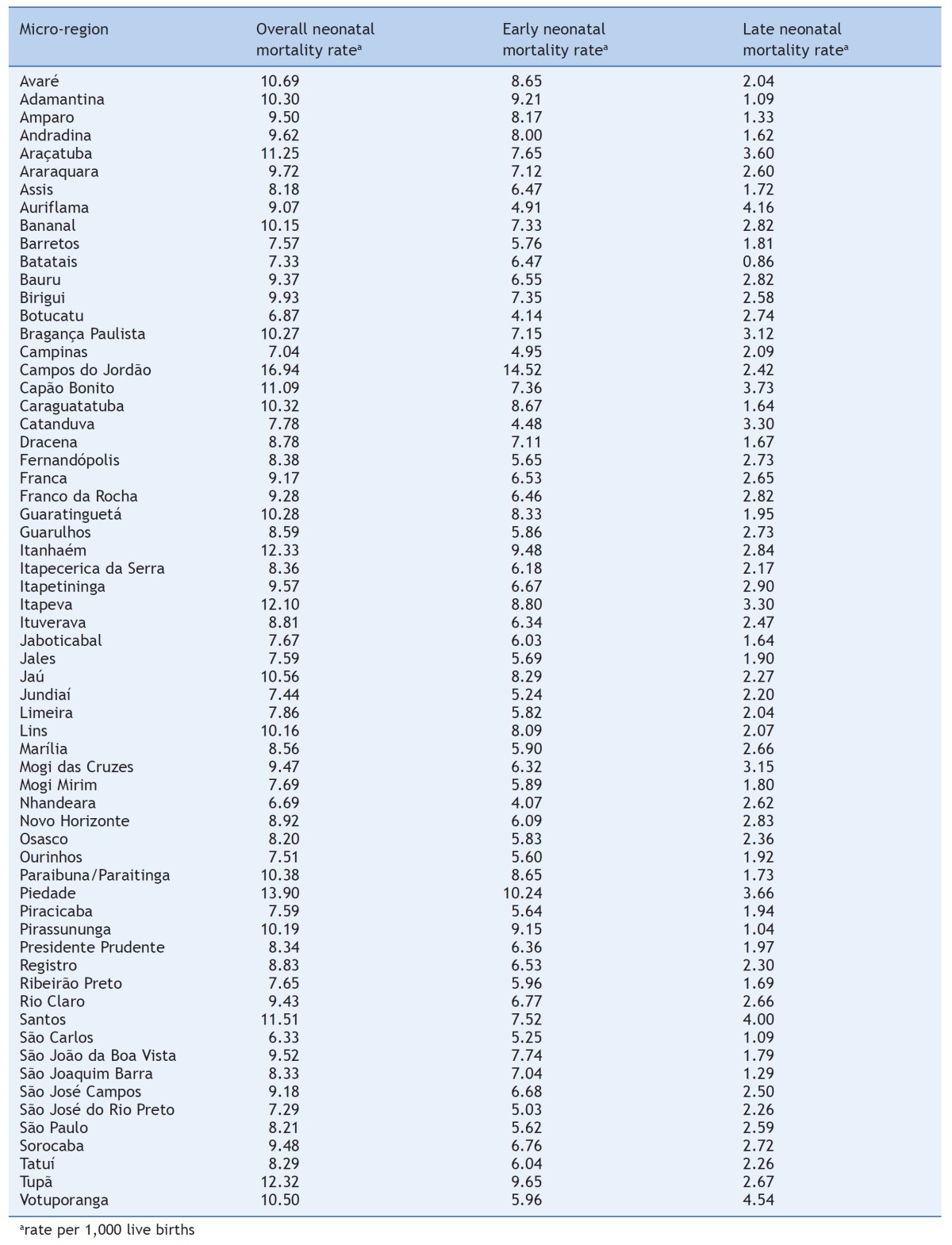
Overall, early, and late neonatal mortality rates according to micro-region, São Paulo, Brazil, 2006-2010.

The Global Moran's index (I) showed statistical significance for the overall neonatal mortality rate (I=0.13), for the early neonatal mortality rate (I=0.15), and for the late neonatal mortality rate (I=0.26). This indicates that the micro-regions form spatial clusters with similar rates.

**Figure 1:**
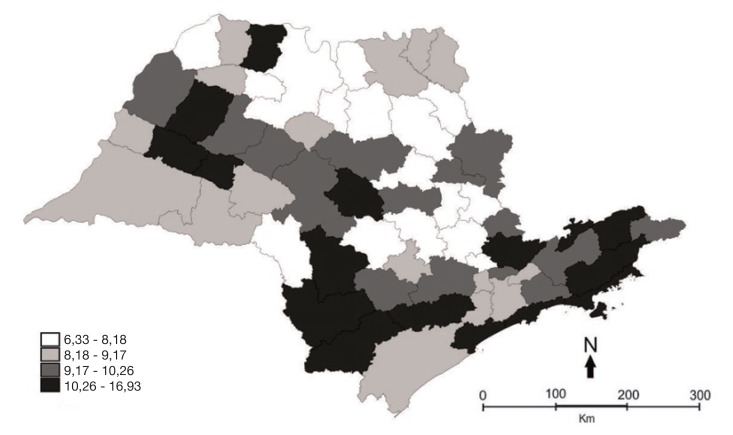
Thematic map of the distribution of the overall neonatal mortality rates per 1,000 live births, according to micro-region, São Paulo, Brazil, 2006-2010

On the thematic map, which shows the overall neonatal mortality rates (Fig. 1), lower mortality rates were observed in the northern and central regions of the state and higher rates in the regions of Vale do Paraiba and the southwest of the state, especially the micro-regions of Capão Bonito, Itapeva, Itanhaém, Santos, Caraguatatuba, Paraibuna/Paraitinga, Campos do Jordão, and Guaratinguetá with the highest rates in the south and southeast regions, and Araçatuba, Adamantina, and Votuporanga in the northwest region. The micro-regions that showed low rates were: São José do Rio Preto, Barretos, Catanduva, Jaboticabal, and Ribeirão Preto in the northern region of the state, and São Carlos, Limeira, Piracicaba, Campinas, and Botucatu in the central and southeast regions.

**Figure 2:**
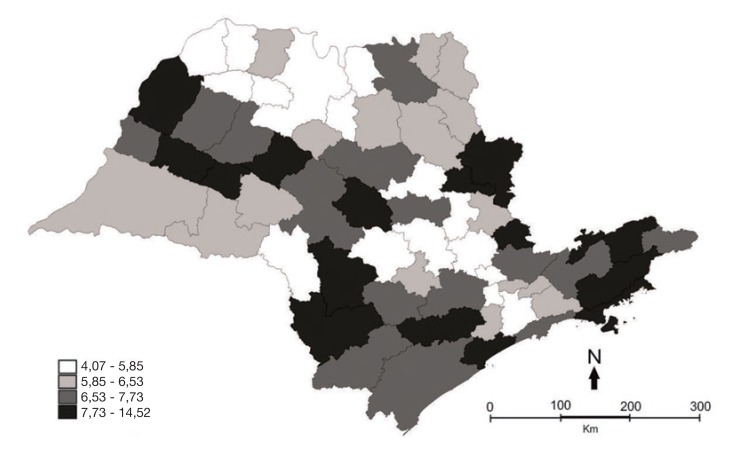
Thematic map of the distribution of the early neonatal mortality rates per 1,000 live births, according to micro-region, São Paulo, Brazil, 2006-2010

Regarding early neonatal mortality, the thematic map (Fig. 2) was similar to the map of overall neonatal mortality rate, especially the micro-regions of Itapeva, Piedade, Itanhaém, Paraibuna/Paraitinga, Caraguatatuba, Guaratinguetá, and Campos do Jordão with the highest rates in the south and southeast regions.

**Figure 3:**
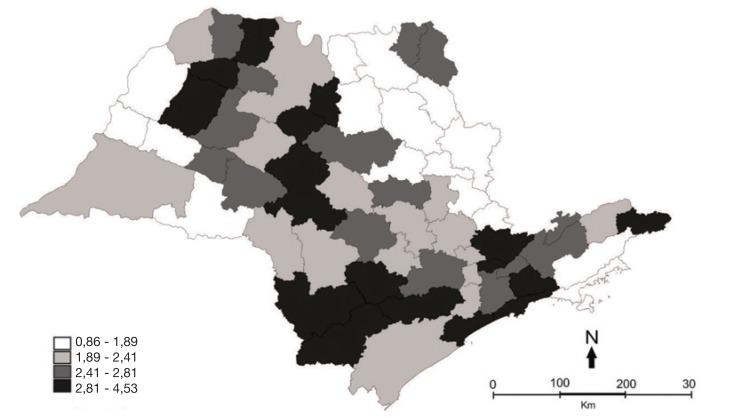
Thematic map of the distribution of the late neonatal mortality rates per 1,000 live births, according to micro-region, São Paulo, Brazil, 2006-2010

Low rates were observed in the micro-regions of Barretos, Jaboticabal, Ribeirão Preto, and São Carlos in the northern and northeastern regions of the state, and Caraguatatuba on the coast. The highest rates are found in the southern and southwestern regions (Fig. 3), especially in the micro-regions of Capão Redondo, Itapeva, Piedade, Itapetininga, Mogi das Cruzes, Santos, Franco da Rocha, and Bananal with the highest rates.

**Figure 4:**
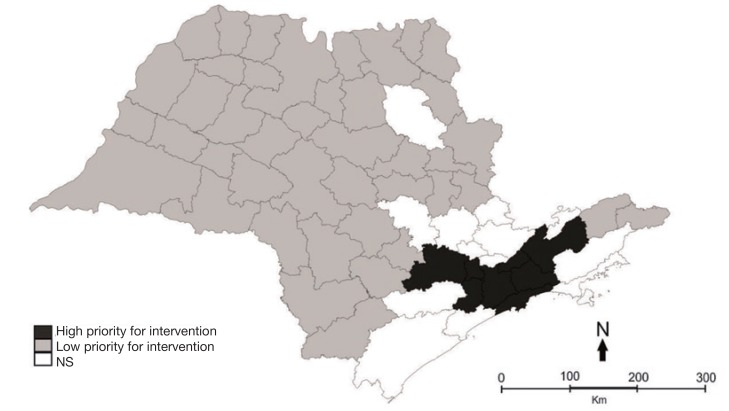
Box map identifying micro-regions of the state of São Paulo, Brazil, according to high, low, and non-significant (NS) priority for intervention related to neonatal mortality, 2006-2010

The box map (Fig. 4) shows the regions with high priority of attention (darker regions): Santos Osasco, Itapecerica da Serra, SãoPaulo, Guarulhos, and Mogi das Cruzes.

## Discussion

This is the first study to spatially analyze neonatal mortality in the state of São Paulo, and it was possible to identify spatial clusters of micro-regions with high rates of neonatal mortality. 

The southern region is one of the poorest in the state;^18^ it is inferred that, in this area, access to good-quality prenatal care can be difficult, the education of the population regarding the care required during pregnancy can be precarious, and hospital care both in the prenatal, as well as in the peri- and postnatal periods may be inadequate.^1^ Thus, these factors may contribute to higher rates of neonatal mortality in the regions of Itapeva, Capão Bonito, Piedade, and Itanhaém, and to moderate rates in Registro.

The Vale do Paraíba region showed high rates of neonatal mortality; a possible explanation is the poor care provided to pregnant women and newborns, as it is a region intersected by a major highway, i.e., the Dutra highway, and it has inpatient and outpatient services. However, it is important to note that these services are concentrated in a few municipalities (São José dos Campos, Taubaté, and Guaratinguetá) and very often the access to these services is hampered due to the distance that must be traveled by the pregnant woman.^19^

The southeastern region of the state also showed micro-regions with high rates of neonatal mortality, where it appears that healthcare access and quality of care are satisfactory; health services are accessible and of good quality, and should be able to identify preventable deaths and implement measures to reduce them. In this situation of high-coverage by the health care system, the inclusion of other criteria or quality markers, which in addition to the minimum of six prenatal care consultations and its start before the 14^th^ week of gestation, should include the request of all routine laboratory tests, obstetric examinations, vaccinations, Pap cervical screening, and recommendations on breastfeeding and childbirth.^6^

It can be observed that socioeconomic factors cannot be solely responsible for the high rate of neonatal mortality, as it appears that the wealthiest micro-regions also had high rates, as can be seen in the thematic map, which shows lower rates of neonatal mortality in the Vale do Ribeira region, which is a region with poor health and development indicators. 

One can speculate that in more developed micro-regions, there are pockets of poverty, which could explain these higher rates or even difficult access to health services; is important to emphasize that the data are related to the mother's residence, thus preventing micro-regions with better health care service, i.e., those centers of referral for pregnant women at risk from other micro-regions, from showing an overestimation of neonatal mortality rates. In the northern region, which showed low rates, these are possibly due to the existence of large referral health centers, such as Ribeirão Preto, Barretos, and São José do Rio Preto, with better care to the sick newborn, as well as better socioeconomic conditions, which allow prenatal care with an earlier onset and a better quality of care for pregnant women in this period.

The difficulty in the access, disorganization, and fragmentation of the health care system and scientific-technical inadequacies of the assistance are difficulties found in the country regarding care for pregnant women and newborns and, in relation to prenatal care, hierarchization and the guarantee of access and quality of care, rather than only the number of consultations, are key in improving attention.^5^^-^ 7 The quality of prenatal and perinatal care is directly related to neonatal death^19^ and even preterm neonates with very low birth weight may survive if given adequate care in the delivery room and good-quality care in the neonatal intensive care unit.^20^

In a case-control study in Fortaleza, neonatal deaths were associated with the quality of prenatal care and the direct assistance during delivery.^1^ Another cohort study in the city of Caxias do Sul showed that despite the low probability of early neonatal death, there were deaths that could have been prevented with better prenatal, perinatal, and postnatal.^2^ In a study conducted in the city of Rio de Janeiro, spatial analysis was used to identify explanatory factors of spatial variations in the rate of early neonatal mortality. The variables that best explain the clusters are "proportion of adolescent mothers," "proportion of individuals living in slums in 1996," and "proportion of heads of the family with income up to one minimum wage."^21^

The present study has limitations inherent to ecological studies, such as cases of possible underreporting or misdiagnosis. The study of the distribution of low birth weight and preterm birth was not carried out, factors that contribute to neonatal mortality.^19^^,^ 22 The level of development of each micro-region was not correlated, in spite of its recognized importance for neonatal mortality, because there are no such data per micro-region, only by municipality. Another possible limitation is the difficulty of assessing the factors associated with late neonatal mortality. Data analysis performed by the Regional Departments of Health (Departamentos Regionais de Saúde DRS) could indicate which DRS should be the subject of more detailed studies; however, there are 17 DRS in the 63 micro-regions of the state of São Paulo, which could create a bias in the results.

Moreover, the study did not consider maternal conditions such as previous diseases and specific complications of pregnancy, which are situations that predispose to hypoxia and perinatal infections and, therefore, often lead to neonatal death. Moreover, no information on maternal hospitalization during pregnancy that could contribute to neonatal death was evaluated, because these data were not available, as they were not included in the databases.

The results of this study corroborate others,^1^^,^ 2 ^,^ 5 ^-^ 7 ^,^ 19 ^,^ 20 ^,^ 22 which reported that the early neonatal mortality rate is the largest component of the overall neonatal mortality rate and that newborns from regions with unfavorable socioeconomic status are at higher risk of neonatal death. These studies indicate that the quality of care provided by health services has to improve in the poorest regions of the state, but in regions with more favorable socioeconomic status, it is necessary that the service provided be effective. The rates of overall, early, and late neonatal mortality in this study are lower than most rates seen in other states of Brazil, except three, Santa Catarina, Rio Grande do Sul, and the Federal District.^4^

The results shown in this study provide assistance for regional and local managers to implement policies to lower rates of neonatal mortality in the state of São Paulo.
