# Supplementary material for: Spatial analysis of neonatal mortality in the state of São Paulo, 2006-2010
Source: Rev Paul Pediatr. 2014 Dec;32(4):374–80. doi: 10.1016/j.rpped.2014.01.001 (PMC4311792; doi:10.1016/j.rpped.2014.01.001)
Supplement: Translation

# Translation: Análise espacial da mortalidade neonatal no estado de São Paulo, 2006-2010☆

**Authors:** Milena Cristina Silva Almeida, Camila Moraes Santos Gomes, Luiz Fernando Costa Nascimento

**Affiliations:** Departamento de Medicina, Universidade de Taubaté (UNITAU), Taubaté, SP, Brasil

**Keywords:** Mortalidade neonatal, Epidemiologia, Sistemas de informação geográfica, Análise espacial

## Abstract

**OBJETIVO:**

Identificar padrões espaciais da distribuição da mortalidade neonatal total, precoce e tardia no Estado de São Paulo.

**MÉTODOS:**

Estudo ecológico e exploratório realizado em microrregiões do estado de São Paulo. Foram obtidos do Departamento de Informações e Informática do Sistema Único de Saúde (Datasus) dados sobre mortalidade neonatal total, precoce e tardia no estado de São Paulo entre 2006 e 2010. Foram calculadas as taxas de mortalidade por 1000 nascidos vivos (NV). Calcularam-se os índices de Moran global dessas taxas e construídos mapas temáticos; foi construído o *Box Map* para identificar microrregiões com alta prioridade de intervenção. A análise espacial foi realizada utilizando o programa computacional TerraView 4.2.1.

**RESULTADOS:**

As taxas de mortalidade neonatal precoce e tardia, foram respectivamente de 6,2/1000 NV e 2,5/1000 NV. Os índices de Moran global (I) foram I=0,13, I=0,15 e I=0,26 para as taxas de mortalidade neonatal total, precoce e tardia, respectivamente, todos com *p*-valor <0,05. Houve aglomerados de microrregiões com altas taxas localizados no sudoeste e leste do estado.

**CONCLUSÃO:**

Com os resultados das análises obtidas neste estudo é possível aos gestores de saúde a implantação de políticas para redução da mortalidade neonatal.

## Introdução

A mortalidade neonatal (óbitos entre 0 e 27 dias de vida) é um importante indicador de saúde de uma população e corresponde a aproximadamente 2/3 dos óbitos infantis; categoriza-se em precoce, que é aquela que ocorre até o sétimo dia incompleto de vida, e tardia, que ocorre entre o sétimo e 28º dia incompleto de vida. A taxa de mortalidade neonatal é composta pelas taxas de mortalidade neonatal precoce e tardia sendo, a primeira, o principal componente e que reflete a assistência de saúde prestada à gestante no período pré-parto, no parto e também a assistência ao recém-nascido logo após o nascimento e nas unidades neonatais.^1^^,^ 2

O óbito neonatal é o principal componente da taxa de mortalidade infantil no Brasil e foi de 9,7/1000 NV em 2010, maior que a de outros países como os EUA (4/1000 NV), Chile (5/1000 NV) e Canadá (4/1000 NV), entre outros, como reportados pela Organização Mundial da Saúde).^3^^,^ 4 No período entre 2001 e 2010, a queda na mortalidade neonatal foi de cerca de 25%.^4^

A mortalidade neonatal pode ser determinada por diversos fatores tais como baixo e extremo baixo peso, prematuridade, malformações congênitas complexas e asfixia neonatal e também por má qualidade na atenção no pré-natal, além de fatores sociodemográficos e iniquidades regionais.^1^^,^ 2 ^,^ 5 ^-^ 8 Entretanto, as causas maternas, na maioria evitáveis, são as mais frequentes causas básicas e os principais desencadeantes da mortalidade neonatal nos países emergentes.^9^^,^ 10 Sabe-se, também, que a maior parte dos óbitos neonatais ocorre em regiões com baixa renda, sendo que crianças que nascem em regiões pobres apresentam maior risco de morte.^10^^,^ 11

A localização espacial dos eventos em saúde e os Sistemas de Informações Geográficas (SIG) têm sido utilizados com mais frequência na área da saúde pública.^12^ Estudo de análise espacial das taxas óbito neonatal no Vale do Paraíba permitiu identificar municípios prioritários para intervenção.^13^

A análise da distribuição espacial da mortalidade neonatal pode oferecer subsídios para ações de melhorias do serviço de saúde com vistas à diminuição dessa taxa de mortalidade. Assim, o objetivo do estudo foi o de identificar padrões espaciais da distribuição da mortalidade neonatal total, precoce e tardia no Estado de São Paulo no período de 2006 a 2010.

## Método

Trata-se de um estudo ecológico e exploratório com dados sobre mortalidade neonatal nas 63 microrregiões do Estado de São Paulo, Brasil, obtido do Departamento de Informações e Informática do Sistema Único de Saúde (Datasus)^14^ no período compreendido entre os anos 2006 a 2010. São Paulo é estado mais populoso do Brasil com cerca de 41 milhões de habitantes. Os dados sobre nascidos vivos foram obtidos do Sistema de Informações sobre Nascidos Vivos - SINASC.^15^

Foi construído um banco com todos os casos de óbito neonatal, calculou-se a taxa de mortalidade neonatal total por 1000 nascidos vivos, a taxa de mortalidade neonatal precoce (aquela que ocorre até o sétimo dia de vida incompleto) e tardia (aquela que ocorre entre o sétimo e 28^o^ dia de vida incompleto), por microrregião do estado de São Paulo. As taxas se referem a todos os óbitos ocorridos no período neonatal em razão de todos os nascimentos do período de 2006 a 2010.

Foi utilizado o programa TerraView 4.2.1 de acesso público e desenvolvido pelo Instituto Nacional de Pesquisas Espaciais (INPE)^16^ para a análise espacial das 63 microrregiões do estado de São Paulo. Foi estimada a autocorrelação espacial das taxas de mortalidade neonatal pelo índice global de Moran (I). O Índice de Moran é utilizado para identificar aglomerados de áreas com riscos semelhantes para ocorrência do desfecho e pode variar de -1 a +1, sendo que os valores próximos de zero indicam a inexistência de autocorrelação espacial significativa entre os valores de dada área e áreas vizinhas. Valores positivos indicam que as microrregiões eram parecidas entre si e, valores negativos, que as microrregiões não se pareciam. Este índice presta-se para testar a hipótese nula que é de independência espacial; neste caso, seu valor seria zero. Valores positivos (entre 0 e +1) indicam correlação direta e, negativos, (entre 0 e -1) correlação inversa.^17^

Foram construídos os mapas temáticos da taxa de mortalidade total, precoce e tardia, além do *Box Map* obtido pelas informações do diagrama de espalhamento de Moran,^17^ que é uma maneira adicional de visualizar a dependência espacial. É dividido em quatro quadrantes: Q1 (valores positivos, médias positivas) e Q2 (valores negativos, médias negativas): indicam pontos de associação espacial positiva, no sentido de que uma localização possui vizinhos com valores semelhantes; Q3 (valores positivos, médias negativas) e Q4 (valores negativos, médias positivas): indicam pontos de associação espacial negativa, no sentido de que uma localização possui vizinhos com valores distintos. 

No *Box Map* as microrregiões localizada em Q1 requerem uma atenção especial com o objetivo de diminuírem as taxas do desfecho estudado, no nosso caso, a mortalidade neonatal. As microrregiões localizadas no Q2 têm baixa prioridade de atenção, pois apresentam taxas baixas.^17^

O nível de significância (α) adotado foi de 5%. Este projeto foi aprovado pelo Comitê de Ética da Universidade de Taubaté número 045/11. 

## Resultados

No período de 2006 a 2010 foram registrados, no estado de São Paulo, 3.000.158 nascidos vivos, houve 18.448 óbitos neonatais precoces (6,2/1000 NV), 7.510 óbitos neonatais tardios (2,5/1000 NV). A tabela 1 mostra os valores das taxas de mortalidade neonatal total, precoce e tardia nas microrregiões do estado de São Paulo.

**Tabela 1:** Taxas de mortalidades neonatais total, precoce e tardia, segundo microrregiões, São Paulo, 2006-2010

Nos coeficientes de Moran Global (I) houve significância estatística para a taxa de mortalidade neonatal total (I= 0,13), para taxa de mortalidade neonatal precoce (I=0,15) e para a taxa de mortalidade neonatal tardia (I=0,26). Isto indica que as microrregiões formam aglomerados espaciais com taxas semelhantes. 

No mapa temático com as taxas de mortalidade neonatal total (fig. 1) foram encontrados valores menores na região norte e central do Estado e, maiores, nas regiões do Vale do Paraíba e sudoeste do Estado, destacando-se as microrregiões de: Capão Bonito, Itapeva, Itanhaém, Santos, Caraguatatuba, Paraibuna/Paraitinga, Campos do Jordão e Guaratinguetá com as maiores taxas nas regiões sul e sudeste, e Araçatuba, Adamantina e Votuporanga na região noroeste. As microrregiões que apresentaram baixas taxas foram: São José do Rio Preto, Barretos, Catanduva, Jaboticabal e Ribeirão Preto no norte do estado e São Carlos, Limeira, Piracicaba, Botucatu e Campinas nas regiões central e sudeste. 

**Figura 1:** Mapa temático da distribuição das taxas de mortalidade neonatal total, por 1000 nascidos vivos, segundo microrregiões, São Paulo, 2006-2010

Para a mortalidade neonatal precoce, o mapa temático (fig. 2) foi semelhante ao mapa da taxa de mortalidade neonatal total, destacando-se as microrregiões de: Itapeva, Piedade, Itanhaém, Paraibuna/Paraitinga, Caraguatatuba, Guaratinguetá e Campos do Jordão com as maiores taxas nas regiões sul e sudeste.

**Figura 2:** Mapa temático da distribuição das taxas de mortalidade neonatal precoce, por 1000 nascidos vivos, segundo microrregiões, São Paulo, 2006- 2010

Com baixas taxas destacam-se as microrregiões de Barretos, Jaboticabal, Ribeirão Preto e São Carlos nas regiões norte e nordeste do estado, e Caraguatatuba no litoral. As maiores taxas se encontram nas regiões sul e sudoeste (fig. 3), destacando-se as microrregiões de Capão Bonito, Itapeva, Piedade, Itapetininga, Mogi das Cruzes, Santos, Franco da Rocha e Bananal com as maiores taxas. 

**Figura 3:** Mapa temático da distribuição das taxas de mortalidade neonatal tardia, por 1000 nascidos vivos, segundo microrregiões, São Paulo, 2006-2010

O Box Map (fig. 4) mostra as regiões com alta prioridade de atenção(regiões mais escuras): Santos, Osasco, Itapecerica da Serra, São Paulo, Guarulhos e Mogi das Cruzes.

**Figura 4:** Box Map identificando microrregiões do estado de São Paulo, segundo prioridade de intervenção Alta, Baixa e não-significante (NS) para mortalidade neonatal, 2006-2010

## Discussão

Este é o primeiro estudo a analisar espacialmente a mortalidade neonatal no Estado de São Paulo, tendo sido possível identificar aglomerados espaciais de microrregiões com taxas elevadas de mortalidade neonatal. 

A região sul é uma das mais pobres do Estado,^18^ infere-se que nessa área o acesso ao pré-natal de qualidade pode ser difícil, a educação da população acerca dos cuidados que se deve ter durante a gestação pode ser precária e a assistência hospitalar tanto no período pré-natal, como no peri e pós-natal pode ser inadequada.^1^ Desta forma, tais fatores podem contribuir para taxas mais altas de mortalidade neonatal nas microrregiões de Itapeva, Capão Bonito, Piedade e Itanhaém e moderadas taxas em Registro.

A região do Vale do Paraíba apresentou elevadas taxas de mortalidade neonatal e uma possível explicação seria o atendimento insatisfatório dispensado à gestante e ao recém-nascido, já que se trata de uma região cortada por uma rodovia importante, a rodovia Dutra, e **que** conta com serviços hospitalares e ambulatoriais. Porém, é importante salientar que esses serviços se concentram em poucos municípios (São José dos Campos, Taubaté e Guaratinguetá) e, muitas vezes, o acesso a eles é prejudicado por conta da distância a ser percorrida pela gestante.^19^

Na região sudeste do Estado, também foram encontradas microrregiões com elevadas taxas de mortalidade neonatal, onde supostamente o acesso à saúde e a qualidade do atendimento são satisfatórios, os serviços de saúde são acessíveis e de boa qualidade e deveriam ser capazes de identificar as mortes evitáveis e implementar medidas para reduzi-las. Nesta situação de alta cobertura de rede assistencial de saúde ,é necessária a inclusão de outros critérios ou marcadores de qualidade, que, além do mínimo de seis consultas de cuidado pré-natal e seu início antes da 14ª semana de gestação, incluiria a solicitação de todos os exames laboratoriais de rotina, a realização de exame obstétrico, a vacinação, o exame citopatológico do colo uterino e a orientações com relação à amamentação e ao parto.^6^

Observa-se que fatores socioeconômicos podem não ser os únicos responsáveis pela alta taxa de mortalidade neonatal, já que microrregiões supostamente mais ricas também apresentaram elevadas taxas, como pode ser visto pelo mapa temático que mostra taxas de mortalidade neonatal menores na região do Vale do Ribeira, que é uma região com indicadores ruins de saúde e desenvolvimento; pode ser pensado que nas microrregiões mais desenvolvidas haja bolsões de pobreza, o que poderia explicar estas taxas maiores ou mesmo dificuldades de acesso aos serviços de saúde; é importante salientar que os dados se referem à residência da mãe, evitando desta forma, que microrregiões com melhores condições de atendimento, isto é, por serem polos de recepção de gestantes de risco de outras microrregiões apresentem superestimação das taxas de mortalidade neonatal.Já na região norte do Estado, que apresentou baixas taxas, estas devem-se possivelmente a existência de grandes centros de referência em saúde, como Ribeirão Preto, Barretos e São José do Rio Preto, com uma melhor atenção aos recém-nascidos enfermos, além de melhores condições socioeconômicas, as quais permitem um pré-natal com início mais precoce e uma melhor condição de atendimento da gestante neste período.

A dificuldade no acesso, a desorganização e a fragmentação do sistema de saúde e inadequações técnico-científicas da assistência são dificuldades encontradas no país quanto à atenção à gestante e ao recém-nascido, sendo que em relação ao pré-natal, a hierarquização, a garantia do acesso e a qualidade do atendimento, e não apenas a quantidade de consultas, são pontos-chave na melhoria da atenção.^5^^-^ 7 A qualidade da assistência pré-natal e perinatal está diretamente relacionada ao óbito neonatal^19^ e mesmo neonatos prematuros de muito baixo peso podem não morrer se bem atendidos na sala de parto e se submetidos a cuidados em unidade de terapia intensiva neonatal.^20^

Em estudo caso-controle em Fortaleza, as mortes neonatais foram associadas à qualidade da assistência pré-natal e da assistência direta ao trabalho de parto^1^. Outro estudo de coorte no município de Caxias do Sulmostrou que, apesar da probabilidade de morte neonatal precoce ter sido baixa, houve mortes que poderiam ter sido evitadas com um melhor atendimento no pré-natal, no parto e na assistência ao recém-nascido.^2^ Em um estudo realizado no município do Rio de Janeiro, a análise espacial foi utilizada para identificar fatores explicativos das variações espaciais da taxa de mortalidade neonatal precoce. As variáveis que melhor explicaram os aglomerados espaciais foram:"proporção de mães adolescentes", "proporção de pessoas residentes em favelas em 1996" e "proporção de chefes com rendimento até um salário mínimo".^21^

O presente estudo apresenta limitações próprias dos estudos ecológicos, como casos de possíveis subnotificações ou erros de diagnóstico. Não foi realizado estudo da distribuição de baixo peso e prematuridade, fatores que contribuem para a mortalidade neonatal.^19^^,^ 22 O nível de desenvolvimento de cada microrregião não foi relacionado, apesar da reconhecida importância deste na mortalidade neonatal, porque não existem estes dados por microrregião, apenas por município. Outra possível limitação foi a dificuldade de avaliar fatores associados à mortalidade neonatal tardia. A análise de dados realizada por Departamentos Regionais de Saúde (DRS) poderia apontar qual DRS deveria ser o alvo de estudos mais detalhados; no entanto, são 17 DRS no estado de São Paulo e 63 microrregiões, o que poderia enviesar os resultados. Além disso, não foram consideradas no estudo as condições maternas, tais como doenças prévias e complicações específicas da gestação, que são situações que predispõem à hipóxia e às infecções perinatais e, consequentemente, costumam levar ao óbito neonatal. Também não foram avaliadas informações sobre internações maternas na gestação que pudessem contribuir para o óbito neonatal porque os dados não estavam disponíveis, por não estarem incluídos nos bancos de dados.

Os resultados deste estudo corroboram com outras pesquisas^1^^,^ 2 ^,^ 5 ^-^ 7 ^,^ 19 ^,^ 20 ^,^ 22 que observaram ser a a taxa de mortalidade neonatal precoce o maior componente da taxa de mortalidade neonatal e que recém-nascidos de regiões com condições socioeconômicas desfavoráveis apresentam maior risco de morte neonatal. Tais estudos indicam que a qualidade da atenção oferecida pelos serviços de saúde tem que melhorar nas regiões mais pobres do Estado, mas, nas regiões mais favorecidas, é necessário que o serviço acessível prestado seja efetivo. As taxas de mortalidades neonatal total, precoce e tardia neste estudo estão abaixo da maioria dos valores de outros estados do Brasil, exceto três que foram Santa Catarina, Rio Grande do Sul e Distrito Federal.^4^

Os resultados apresentados neste estudo dão subsídios para os gestores regionais e locais para a implementação de políticas voltadas para a diminuição das taxas de mortalidade neonatal no estado de São Paulo.
